# Statistical analysis plan for the ‘Tranexamic acid for hyperacute primary IntraCerebral Haemorrhage’ (TICH-2) trial

**DOI:** 10.1186/s13063-017-2341-5

**Published:** 2017-12-20

**Authors:** Katie Flaherty, Philip M. Bath, Robert Dineen, Zhe Law, Polly Scutt, Stuart Pocock, Nikola Sprigg, on behalf of the TICH-2 investigators

**Affiliations:** 1 Stroke Trials Unit, Division of Clinical Neuroscience, University of Nottingham, City Hospital Campus, Hucknall Road, Nottingham, NG5 1PB UK; 2 Imaging Sciences, Division of Clinical Neuroscience, University of Nottingham, Nottingham, UK; 3 Department of Medical Statistics, London School of Hygiene and Tropical Medicine, London, UK; 4 Department of Medicine, National University of Malaysia, Kuala Lumpur, Malaysia

**Keywords:** Hyperacute, Spontaneous intracerebral haemorrhage, Tranexamic acid, Randomised trial, Placebo-controlled

## Abstract

**Rationale:**

Aside from blood pressure lowering, treatment options for intracerebral haemorrhage remain limited and a proportion of patients will undergo early haematoma expansion with resultant significant morbidity and mortality. Tranexamic acid (TXA), an anti-fibrinolytic drug, has been shown to significantly reduce mortality in patients, who are bleeding following trauma, when given rapidly. TICH-2 is testing whether TXA is effective at improving outcome in spontaneous intracerebral haemorrhage (SICH).

**Methods and design:**

TICH-2 is a pragmatic, phase III, prospective, double-blind, randomised placebo-controlled trial. Two thousand adult (aged ≥ 18 years) patients with an acute SICH, within 8 h of stroke onset, will be randomised to receive TXA or the placebo control. The primary outcome is ordinal shift of modified Rankin Scale score at day 90. Analyses will be performed using intention-to-treat.

**Results:**

This paper and its attached appendices describe the statistical analysis plan (SAP) for the trial and were developed and published prior to database lock and unblinding to treatment allocation. The SAP includes details of analyses to be undertaken and unpopulated tables which will be reported in the primary and key secondary publications. The database will be locked in early 2018, ready for publication of the results later in the same year.

**Discussion:**

The SAP details the analyses that will be done to avoid bias arising from prior knowledge of the study findings. The trial will determine whether TXA can improve outcome after SICH, which currently has no definitive therapy.

**Trial registration:**

ISRCTN registry, ID: ISRCTN93732214. Registered on 17 January 2013.

**Electronic supplementary material:**

The online version of this article (doi:10.1186/s13063-017-2341-5) contains supplementary material, which is available to authorized users.

## Background

Haemorrhagic stroke or intracerebral haemorrhage (ICH), caused by bleeding in the brain, can be devastating and is a common cause of death and disability, both in the UK and worldwide. Despite development of effective treatments for ischaemic stroke (thrombolysis, aspirin, hemicraniectomy, thrombectomy) there is no proven effective treatment for ICH. Outcome after ICH is closely related to whether brain bleeding expands after onset, so-called haematoma expansion, or whether re-bleeding occurs; both are associated with a poor outcome (death and disability) [1]. Haematoma expansion is related to both haemostatic factors and blood pressure; furthermore, haematoma volume can be reduced surgically. For ICH, aside from intensive blood pressure lowering, treatment options remain limited and a proportion of patients will undergo early haematoma expansion with resultant significant morbidity and mortality. An ICH that occurs in the absence of a known underlying cause, e.g. aneurysm, arteriovenous malformation (AVM) or tumour, is referred to as a spontaneous intracerebral haemorrhage (SICH).

Tranexamic acid (TXA) is a licensed anti-fibrinolytic drug that can be administered intravenously or orally and is used in a number of bleeding conditions to reduce bleeding [2, 3]. It has been tested in aneurysmal subarachnoid haemorrhage, where it reduced the risk of re-bleeding at the expense of increased risk of cerebral ischaemia [4]; however, administration was for a week, conferring prolonged exposure to risk of ischaemic events. In a recent mega-trial (CRASH-2) in 20,000 patients, with major bleeding following trauma, TXA significantly reduced mortality, odds ratio (OR) 0.91 (0.85, 0.97), with no increase in vascular occlusive events [5]. Treatment was most effective when given rapidly; delayed administration was associated with lack of efficacy and potential harm [6]. In another randomised controlled trial in traumatic ICH, TXA showed trends towards reduced death: OR 0.69 (0.35, 1.39), and death or dependency: OR 0.76 (0.46, 1.27), without increased thromboembolic events [7]. Additionally, TXA has been found to restrict haematoma expansion in acute SICH in a small non-randomised study, although this did not report on safety [8]. In another small study (*n* = 156), rapid administration of a bolus of TXA within 24 h of stroke was observed to reduce haematoma expansion (17.5% vs. 4.3%) [9]. In this study, TXA was given in combination with intensive blood pressure control, suggesting that it may be possible to combine haemostatic and haemodynamic approaches. There have been recent calls in the academic literature for large clinical trials to examine the use of TXA in ICH [10].

The on-going TICH-2 trial is a phase III, prospective, double-blind, randomised placebo-controlled trial, which will assess whether TXA (intravenous 1 g bolus, 1 g infusion/8 h) in acute (<8 h) SICH is safe and reduces death or dependency (measured as a shift in the modified Rankin Scale (mRS) score). TICH-2 is pragmatic in design, with minimal inclusion and exclusion criteria. Further information regarding randomisation can be found in the protocol [11]. TICH-2 followed a two-phase design: the 12-month start-up phase (to assess feasibility) aimed to recruit a minimum of 300 participants from 30 centres, then the main phase is continuing to recruit to a target of 2000 participants from 120 centres over 43 months. The Independent Data Monitoring Committee (iDMC) assessed the trial every 6 months and recommended that the trial should continue on each occasion. Whilst recruitment in the UK was ahead of schedule, recruitment internationally was delayed by prolonged approval processes. Therefore, a 12-month, no-cost extension was sought and approved in April 2016 to allow additional time to enable the trial to reach 2000 participants. Recruitment rate subsequently increased, with recruitment likely to exceed the original 2000 participant target. The Trial Steering Committee recommended that recruitment continues until the end of the allotted funding in order to maximise recruitment, international participants, recruitment into the magnetic resonance imaging (MRI) sub-study and statistical power.

## Methods and design

The statistical analysis plan (SAP), detailed in Additional file 1, will ensure that the analyses are not data driven or selectively reported. Presentation of primary analyses is expected in spring 2018, after all participants have been followed up to day 90. Additional file 1 will detail tables to be included in the primary publication, as well as brief descriptions of other planned publications.

The primary outcome, death or dependency (ordinal shift on mRS) at day 90, will be analysed by intention-to-treat using ordinal logistic regression (OLR), with adjustment for minimisation factors. Whilst there are limitations to using an ordinal analysis of the mRS score (for example, moving from a 6 to a 5 may not be perceived as clinically useful and distances between points are not always equivalent), this approach is widely accepted across stroke trials and recommended by the European Stroke Organisation (ESO) Outcomes Working Party [12]. A number of secondary and sub-group analyses will be undertaken; time to randomisation is likely to be the most important sub-group and so will be examined in more detail.

The results will determine whether TXA can improve outcome after SICH, which currently has no proven therapy. Three sub-studies are being run in conjunction with the trial; one for a telephone follow-up at day 365, one which takes two MRI scans at days 5 and 90 and the third one is a single-centre sub-study that explores plasma biomarkers at baseline, day 2, and day 7. A number of secondary publications are also planned, including; a paper giving further details on randomisation and baseline characteristics and a number of papers addressing secondary outcomes. An individual patient data meta-analysis is planned; using data from studies that looked at using TXA for ICH.

### Data sharing

Once completed, data from TICH-2 will be added to summary and individual patient data meta-analyses in acute stroke. IPD will be made available to the ‘Virtual International Stroke Trials Archive’ (VISTA) [13], and subsequently over the web, as with the International Stroke Trial [14]. Similarly, anonymised baseline and on-treatment neuroimaging data will be published [15].

### Supporting information

Additional supporting information may be found in the online version of this article. Additional file 1 shows the SAP (TICH-2).

#### Trial status

At the time of first submission the TICH-2 trial was ongoing with recruitment due to finish on 30 September 2017 and with follow-up of the primary outcome ongoing until 30 December 2017.

## Additional file

Additional file 1: ‘TICH-2 statistical analysis plan (SAP) appendix V1.6.doc’ includes: Appendix A – SAP; Appendix B – definitions; Appendix C – tables and figures; Appendix D – secondary publications. (DOCX 611 kb)

## Abbreviations

BLR: Binary logistic regression
CPHR: Cox proportional hazards regression
CT: Computed tomography
EQ-5D HUS: EuroQol 5-dimensions Health Utility Status
EQ-VAS: EuroQol Visual Analogue Scale
GCS: Glasgow coma scale
HE: Haematoma expansion
HR: Hazard ratio
HV: Haematoma volume
ICH: Intracerebral haemorrhage
IHD: Ischaemic heart disease
IQR: Interquartile range
ITT: Intention-to-treat
IVH: Intraventricular haemorrhage
MD: Mean difference
MLR: Multiple linear regression
mRS: modified Rankin Scale
NIHSS: National Institutes of Health Stroke Scale
OCSP: Oxfordshire Community Stroke Project classification
OLR: Ordinal logistic regression
OR: Odds ratio
SAE: Serious adverse event
SAP: Statistical analysis plan
SBP: Systolic blood pressure
SD: Standard deviation
SICH: Spontaneous intracerebral haemorrhage
TIA: Transient ischaemic attack
TICS-M: Telephone Interview for Cognitive Status-M
ZDS: Zung Depression Scale
