# Supplementary material for: Statistical analysis plan for the ‘Tranexamic acid for hyperacute primary IntraCerebral Haemorrhage’ (TICH-2) trial
Source: Trials. 2017 Dec 20;18:607. doi: 10.1186/s13063-017-2341-5 (PMC5738041; doi:10.1186/s13063-017-2341-5)
Supplement: Additional file 1: — ‘TICH-2 statistical analysis plan (SAP) appendix V1.6.doc’ includes: Appendix A – SAP; Appendix B – definitions; Appendix C – tables and figures; Appendix D – secondary publications. (DOCX 611 kb) [file 13063_2017_2341_MOESM1_ESM.docx]

**STATISTICAL ANALYSIS PLAN**

**Tranexamic acid for hyperacute primary IntraCerebral Haemorrhage (TICH-2) trial**

Katie S Flaherty, Philip M Bath, Stuart Pocock, Robert Dineen, Zhe Kang Law, Polly Scutt, Nikola Sprigg, on behalf of the TICH-2 investigators

Author for correspondence:

Nikola Sprigg

Stroke Trials Unit, Division of Clinical Neurosciences, University of Nottingham, City Hospital campus, Hucknall Road, Nottingham NG5 1PB UK

Tel: +44 115 823 1778

Fax: +44 115 823 1767

E-mail: [nikola.sprigg@nottingham.ac.uk](mailto:nikola.sprigg@nottingham.ac.uk)


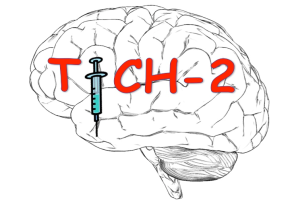


Contents

[Abbreviations 4](#_Toc464210164)

[Appendix A 5](#_Toc464210165)

[1 Introduction 5](#_Toc464210166)

[2 Primary research question 5](#_Toc464210167)

[3 Trial design 5](#_Toc464210168)

[3.1 Design 5](#_Toc464210169)

[3.2 Trial population 5](#_Toc464210170)

[3.3 Randomisation 5](#_Toc464210171)

[3.4 Minimising bias 6](#_Toc464210172)

[3.5 Ethics and regulatory approval 6](#_Toc464210173)

[4 Statistical Analysis Plan (SAP) 6](#_Toc464210174)

[4.1 Introduction 6](#_Toc464210175)

[4.2 Primary outcome 6](#_Toc464210176)

[4.3 Subgroup analyses 7](#_Toc464210177)

[4.4 Secondary outcomes 8](#_Toc464210178)

[4.5 Safety analyses 8](#_Toc464210179)

[4.6 Missing data 8](#_Toc464210180)

[4.7 Analysis populations 9](#_Toc464210181)

[4.8 Sample size 9](#_Toc464210182)

[4.9 Compliance 9](#_Toc464210183)

[4.10 Analysis methods 9](#_Toc464210184)

[Appendix B – Definitions 11](#_Toc464210185)

[1 Definitions of events/ outcomes 11](#_Toc464210186)

[Acute coronary syndrome (ACS) 11](#_Toc464210187)

[2 Other definitions 12](#_Toc464210188)

[2.1 Protocol violations 12](#_Toc464210189)

[2.2 Oxfordshire Community Stroke Project (OCSP) classification 13](#_Toc464210190)

[2.3 Euroqol-5D 14](#_Toc464210191)

[2.4 Haematoma volume 15](#_Toc464210192)

[Appendix C – Tables and figures in primary publications 16](#_Toc464210193)

[Table 1 – Baseline characteristics 16](#_Toc464210194)

[Table 2 – Compliance 18](#_Toc464210195)

[Table 3 – Outcomes 19](#_Toc464210196)

[Table 4 – Serious adverse events 21](#_Toc464210197)

[Table 5 – Outcome by subgroups 23](#_Toc464210198)

[Figure 1 – Trial flow diagram 24](#_Toc464210199)

[Figure 2 – Shift plot of mRS at day 90 by treatment group 25](#_Toc464210200)

[Appendix D – Secondary publications 26](#_Toc464210201)

[1 Published 26](#_Toc464210202)

[2 Submitted 26](#_Toc464210203)

[3 In preparation 26](#_Toc464210204)

[4 Planned (not a complete list) 26](#_Toc464210205)

[References 28](#_Toc464210206)

### Abbreviations

BLR: Binary logistic regression

CPHR: Cox proportional hazards regression

CT: Computed tomography

EQ-5D HUS: Euroqol 5-dimensions Health Utility Status

EQ-VAS: Euroqol Visual Analogue Scale

GCS: Glasgow coma scale

HE: Haematoma expansion

HR: Hazard ratio

HV: Haematoma volume

ICH: Intracerebral haemorrhage

IHD: Ischaemic heart disease

IQR: Interquartile range

ITT: Intention-to-treat

IVH: Intraventricular haemorrhage

MD: Mean difference

MLR: Multiple linear regression

mRS: modified Rankin Scale

NIHSS: National Institutes of Health Stroke Scale

OCSP: Oxfordshire Community Stroke Project classification

OLR: Ordinal logistic regression

OR: Odds ratio

SAE: Serious adverse event

SAP: Statistical analysis plan

SBP: Systolic blood pressure

SD: Standard deviation

SICH: Spontaneous intracerebral haemorrhage

TIA: Transient ischaemic attack

TICS-M: Telephone Interview for Cognitive Status-M

ZDS: Zung Depression Scale.

# Appendix A

## 1 Introduction

Intracerebral haemorrhage (ICH) remains the most devastating form of stroke, with high early mortality and the majority of survivors remaining disabled; despite advances in management of ischaemic stroke, outcome in ICH has remained static for decades [1]. Around a quarter of ICH are complicated by haematoma expansion (HE) in the first 24 hours and this is associated with poor outcome [2]. Haematoma expansion is related to both haemostatic factors and blood pressure; furthermore, haematoma volume can be reduced surgically. Aside from blood pressure lowering, treatment options for ICH remain limited and a proportion of patients will undergo early haematoma expansion with resultant significant morbidity and mortality.

## 2 Primary research question

TICH-2 aims to test the hypothesis that intravenous tranexamic acid (TXA) is superior to placebo by reducing death or dependency, measured as a shift in modified Rankin Scale (mRS), at day 90 when given within 8 hours of spontaneous ICH (SICH).

## 3 Trial design

### 3.1 Design

TICH-2 is a pragmatic international double-blind randomised placebo-controlled parallel group, phase III trial.

### 3.2 Trial population

Adult (≥18 years) patients with an acute SICH within 8 hours of stroke onset (where stroke onset time is unknown, the time of when last known to be well will be used) are eligible for enrolment; a full list of exclusion criteria can be found in the protocol.

### 3.3 Randomisation

All participants eligible for inclusion will be randomised centrally using a secure internet site in real-time. Randomisation involves stratification by country and minimisation on key prognostic factors, detailed in section 3.3.1, as this approach ensures concealment of allocation, minimises differences in key baseline prognostic variables and slightly improves statistical power [3]. Randomisation will allocate a number corresponding to a treatment pack and the participant will receive treatment from the allocated numbered pack. In the event of computer failure (for example: server failure), investigators will follow the working practice document for computer system disaster recovery, which will allow the participant to be randomised following standardised operating procedure [4]

#### 3.3.1 Minimisation factors

The minimisation criteria are given below, along with their low risk versus high risk values:

- Age, <70 vs. ≥70 years
- Sex, female vs. male
- Time from onset to randomisation, <3 vs. ≥3 hours
- Mean systolic blood pressure, <170 vs. ≥170
- Stroke severity (National Institutes of Health Stroke Scale, NIHSS), <15 or ≥15
- Presence of intraventricular haemorrhage, no vs. yes
- Known history of antiplatelet treatment, used immediately prior to stroke onset, no vs. yes

### 3.4 Minimising bias

Multiple measures are taken to minimise bias; double blinding to treatment, blinded adjudication of scans and SAEs, blinded central telephone assessment of three month outcome by staff at National Coordinating Centres, assessment of patient recall of treatment, analysis by intention-to-treat and adjustment for minimisation factors.

### 3.5 Ethics and regulatory approval

TICH-2 is conducted in accordance with the ethical principles that have their origin in the Declaration of Helsinki, 1996; the principles of Good Clinical Practice, in accordance with the Medicines for Human Use Regulations, Statutory Instrument 2004, 1031 and its subsequent amendments and the Department of Health Research Governance Framework for Health and Social care, 2005. The trial received approval from the Medicines and Healthcare products Regulatory Agency on 23^rd^ October 2012, Research Ethics Committee on 23^rd^ November 2012, and the respective National Health Service Research & Development department on 15^th^ February 2013.

## 4 Statistical Analysis Plan (SAP)

All analyses will compare TXA with the placebo control, unless stated otherwise. All regression analyses will be adjusted for the stratification and minimisation criteria, as outlined in section 3.3.

### 4.1 Introduction

The trial is designed to recruit 2,000 participants in total; 300 from the 12 month start-up phase and a further 1,700 from the main phase. Recruitment will end on 30^th^ September 2017. After follow-up at day 90 and database cleaning, the final dataset will be locked and the trial statisticians unblinded to treatment. When all analyses has finished and been published trial data may be available upon request.

### 4.2 Primary outcome

Death or dependency (ordinal shift on mRS) at day 90 will be compared between TXA and placebo by intention-to-treat using ordinal logistic regression, with adjustment for stratification and minimisation covariates, outlined in section 3.3. The assumption of proportional odds will be tested using the likelihood ratio test. If the assumption of proportional odds does not hold; the lack of proportionality will be highlighted and the OLR will still be used, providing the treatment effect across mRS scores is relatively consistent. If is it not consistent then we will perform multiple linear regression.

### 4.3 Subgroup analyses

Comparisons between the treatments will be performed in pre-specified subgroups, the low risk groups are given first in the brackets, as follows;

- The minimisation criteria
- Computed tomography angiography, CTA (yes, no)
- Haematoma location (deep, lobar)
- Ethnicity (other, white).

The subgroup analysis does not comprise the primary analysis and thus has not informed the sample size calculation. The interpretation of any subgroup effect will be based on interaction tests (i.e. evidence of differential treatment effects in the different subgroups) and there will be no adjustment for multiple testing. The minimisation criteria were chosen and thus also included in the subgroup analysis as they are independent prognostic indicators of ICH.

Time to randomisation is likely to be the most important subgroup. Exploratory analysis of the CRASH-2 trial, which compared TXA to a placebo control in adult trauma patients who had or were at risk of significant bleeding, found that patients who received treatment within three hours of trauma had significantly fewer deaths due to bleeding if they received TXA than those who received placebo. On the other hand, significantly more bleeding deaths were seen for those taking TXA if they were treated after three hours [5]. To explore this subgroup in detail exploratory analysis looking at distributions and plots will be used to examine trends in the data; as well as logistic regression models using both binary (cut at 3 hours and 4.5 hours) and continuous time effects, along with corresponding interactions within these models.

A recent systematic review has found that the spot sign, seen on CTA, is a reliable imaging marker for HE, a known predictor of neurological deterioration [6], although a second review concluded that methodological heterogeneity and potential for bias in studies limited synthesis of the data [7]. Participants with greater risk of HE are most likely to benefit from TXA.

Different haematoma locations can affect outcome in patients with ICH. Pontine, infra-tentorial, large basal ganglionic haematomas and primary intraventricular haemorrhage carried a worse prognosis than supratentorial lobar haematomas, probably due to the risk of herniation syndromes in the former locations [8, 9]. Lobar temporal or temporoparietal haematomas predict worse outcome than frontal or parieto-occipital haematomas. [10] TXA may have a greater beneficial effect on patients with supratentorial lobar haematoma due to their better prognosis.

The incidence of ICH, proportion of strokes that are ICHs and case fatality rate vary according to ethnicity. The incidence of ICH is two times higher in East and Southeast Asian people (51.8/100,000 person-years) compared to the estimated worldwide incidence (24.6/100,000 person-years)[11]. In addition a larger proportion of stroke subtypes are ICHs in East and Southeast Asia (22% to 40.6%) compared to western populations (9% to 18%) [12-15]. The 30-day case fatality of approximately 40% was reportedly similar in different regions worldwide, except for two Japanese studies that reported a much lower 30-day case fatality rate of 13% and 18% [16, 17]. These differences may be caused by environmental or genetic factors [18]. TXA may have different effects on Asians compared to western populations.

### 4.4 Secondary outcomes

Binary logistic regression (BLR) will be used for binary outcomes, including death, SAEs and thromboembolic events. Multiple linear regression (MLR) will be used for continuous measures, including haematoma expansion. Wilcoxon rank sum test will be used for continuous measures which are not normally distributed, including Barthel Index. Cox proportional hazards regression (CPHR) will be used for time to event analyses, including death. All regression analyses will be performed with adjustment for baseline factors, as stated in section 4. To review the overall trend of the data a global test (Wei-Lachin) will be used on a combination of outcome measures [19, 20]. The impact of TXA on quality of life will be assessed using the EuroQoL measure. A full health-economic analysis will only be performed after completion of the main phase of the trial.

### 4.5 Safety analyses

The safety outcomes, as listed in section 4.5.1, will be adjudicated by an independent adjudicator who is blind to treatment allocation. The number of participants with SAEs will be analysed, rather than the total number of SAEs, on the safety population (as defined in section 4.7.1). BLR will be performed on each of the safety outcomes. Definitions of them can be found in appendix B, section 1.

#### 4.5.1 Safety outcomes:

- Death
- Death within 28 days of randomisation
- Acute coronary syndrome (ACS)
- Myocardial infarction (NSTEMI)
- Myocardial infarction (STEMI)
- Ischaemic stroke
- Transient ischaemic attack (TIA)
- Peripheral arterial disease (PAD)
- Deep vein thrombosis (DVT)
- Pulmonary embolism (PE)
- Seizure/ convulsions
- Venous thrombosis (any site)

In addition to the above outcomes, we will assess for any safety interaction between the treatment effect and time to randomisation.

### 4.6 Missing data and death

Any missing data are reported. For participants to be included in the primary analysis they must have their mRS score at day 90 recorded along with values for all of the minimisation criteria; if not then they will be excluded from the analysis. To include as many of the participants as possible we are going to backfill any missing minimisation criteria from their randomisation form. The first step is to contact the recruiting centre and ask if they know have this information, if not then imputation will be used. If any of the individual NIHSS measures are missing then the highest risk value will be imputed to ensure that a total NIHSS can be calculated for each participant. If the history of antiplatelets is not known and cannot be found then the highest risk value will be imputed, as would have been used in the randomisation process. Participants who have died before day 90 will be given death scores for outcome measures; mRS = 6, EQ-5D = 0, Barthel index = -5, EQ-VAS = -1, TICS-M = -1, animal naming = -1, Zung = 102.5. Missing outcome data from other follow-ups will be excluded from any analyses. If a participant has withdrawn consent no further information will be collected; however, data collected thus far will be used for analyses.

### 4.7 Analysis populations

#### 4.7.1 Safety population

All randomised participants.

#### 4.7.2 Intention-to-treat (ITT) population

All randomised participants, with the primary outcome recorded.

### 4.8 Sample size

The null hypothesis (H_0_) is that TXA does not alter death or dependency in participants with acute SICH. The alternative hypothesis (H_A_) is that death or dependency differs between those participants randomised to TXA versus placebo. A total sample size of 2,000 (1,000 per group) participants with acute SICH are required, assuming overall significance (alpha) = 0.05; power (1-beta) = 0.90; distribution in mRS (mRS 0 = 4%, 1 = 17% 2 = 16% 3 = 19% 4 = 24% 5 = 7% 6 [death] = 13%) based on data from participants with primary ICH in the ENOS trial); ordinal odds ratio of 0.79; increases due to losses to follow-up of 5%; and a reduction of 20% for baseline covariate adjustment [21]. In summary, a trial of 2,000 participants will have 90% power to detect an ordinal shift of mRS outcome with odds ratio 0.79.

### 4.9 Compliance

Compliance will be assessed by examining the participant’s drug chart and recording evidence of treatment administration. Compliance will be recorded on the case report forms at end of treatment (day 2).

### 4.10 Analysis methods

Data variables will, in the main, be analysed using BLR, CPHR, OLR or MLR, thereby allowing adjustment for baseline covariates. Cox’s proportional hazards model also takes into account time until the specific event and censored observations. Section 4.10.1 gives some of the outcomes to be analysed and what method will be used for the analysis.

#### 4.10.1 Clinical variables

- BLR: Death; Death or deterioration (day 2/7); Discharged home by day 90
- CPHR: Death; hospital discharge
- OLR: mRS (day 90); discharge disposition
- MLR: NIHSS; Length of stay in hospital; BI; TICS-M; ZDS

#### 4.10.2 Procedures

The primary paper will be analysed using two separate sets of statistical code, both written specifically for the purpose; another set of code, based on the Data Monitoring Committee report, will also be available. This approach will allow the results to be crosschecked. Code for all of the programmes will be written in SAS version 9.3.

# Appendix B – Definitions

## 1 Definitions of events/ outcomes

## Acute coronary syndrome (ACS)

The diagram below will help distinguish between the types of acute coronary syndromes in patients presenting with acute cardiac chest pain:

**
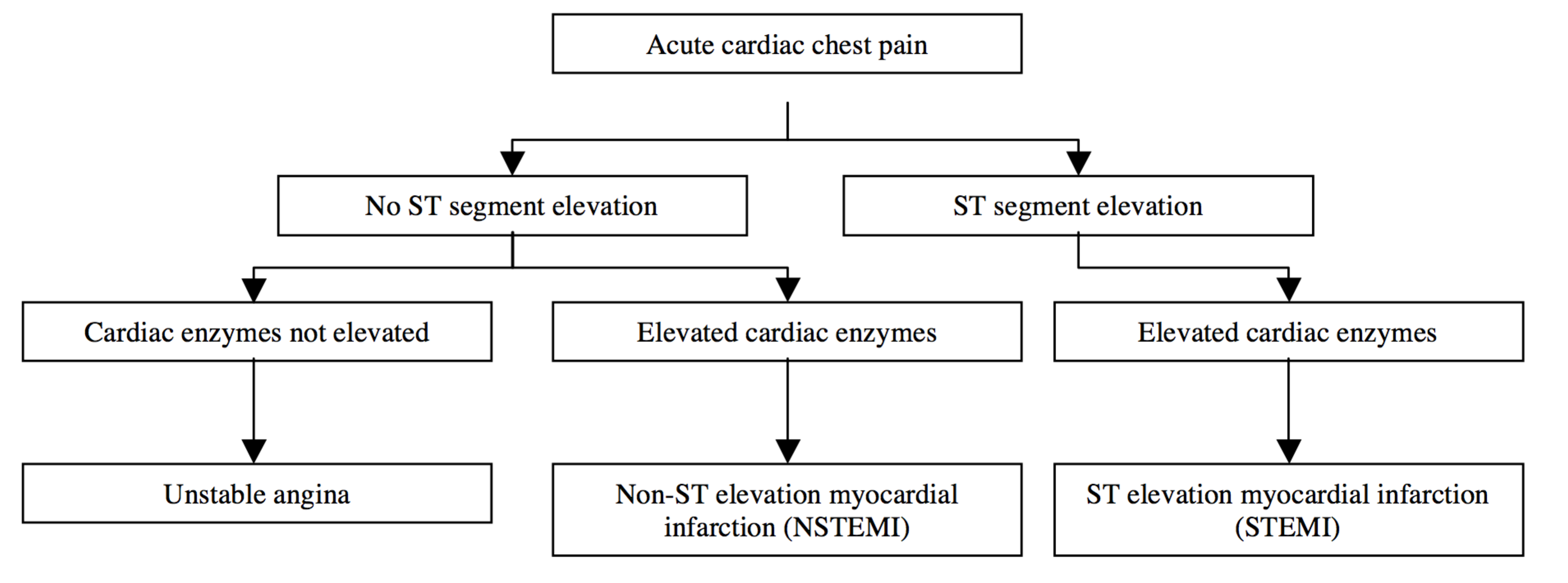
**

**Unstable Angina**

Although there is no universally accepted definition of unstable angina, it has been described as a clinical syndrome between stable angina and acute myocardial infarction.

**Myocardial infarction (MI)**

Either one of the following criteria satisfies the diagnosis for an acute, evolving or recent MI:

1. Typical rise and gradual fall (troponin) or more rapid rise and fall (CK-MB) of biochemical markers of myocardial necrosis with at least one of the following:

- ischaemic symptoms;
- development of pathologic Q waves on the ECG;
- ECG changes indicative of ischemia (ST segment elevation or depression); or
- Coronary artery intervention (e.g. coronary angioplasty).

2. Pathologic findings of an acute MI.

**Ischaemic stroke**

A clinical syndrome characterised by rapidly developing clinical symptoms and/or signs of focal (and at times global) loss of cerebral function with symptoms lasting for more than 24 hours or leading to death, with no apparent cause other than that of vascular origin. Classification as ischaemic is based on results of head CT/MRI imaging excluding haemorrhage.

**Transient ischaemic attack (TIA)**

A sudden focal neurological deficit of the brain or eye, presumed to be of vascular origin and lasts less than 24 hours.

**Peripheral arterial disease (PAD)**

A sudden blockage of a peripheral artery; the blockage may result from a blood clot, embolism, dissection or trauma. Symptoms usually start suddenly. Acute peripheral arterial limb occlusion includes the 7 symptoms listed below:

1. Severe pain
2. Coldness
3. Paraesthesia
4. Loss of sensation
5. Paleness in an extremity
6. Lack of pulse in an extremity
7. Blue skin in affected limb. It can also affect the arteries that carry blood from the kidneys and stomach.

Examples of evidence required;

- Clinical
- Radiological details e.g. angiogram

**Seizure/ convulsions**

Focal or generalised seizures, tonic-clonic seizures or partial seizures; diagnosed clinically after review by an appropriately trained physician.

**Venous thromboembolism (VTE)**

This encompasses both deep vein thrombosis (DVT) and pulmonary embolism (PE).

Examples of evidence required;

Clinical detail and

DVT:

- Ultrasound
- Venography

PE:

- VQ (Ventilation Perfusion) Scan
- CTPA (CT Pulmonary Angiogram) scan

## 2 Other definitions

### 2.1 Protocol violations

A protocol violation is a major deviation from the trial protocol; where a participant is enrolled in spite of not fulfilling all the inclusion and exclusion criteria, or where deviations from the protocol could affect the trial delivery or interpretation significantly.

The following baseline characteristics constitute a protocol violation:

1. Randomisation > 8 hours from onset of symptoms
2. Participant less than 18 years of age
3. Failure to obtain appropriate consent
4. Pre-morbid dependency (mRS) >4
5. Baseline cranial imaging shows underlying structural abnormality such as tumour or arterial venous malformation
6. On anticoagulation
7. Randomising event was secondary to trauma
8. Glasgow Coma Score < 5
9. Known probable life expectancy of less than 3 months
10. Female patient of childbearing potential, pregnant or breastfeeding at randomisation
11. Existing contra-indication to TXA known at the time of randomisation
12. Existing participation in another drug or devices trial, with the exception of the secondary prevention trial, Restart or Stop Antithrombotics Randomised Trial (RESTART). Participants enrolled in TICH-2 may be enrolled in RESTART after 21 days.

The following practice during the trial constitutes a protocol violation:

1. Subsequent randomisation into another drug or devices trial with the exception of the secondary prevention trial, RESTART. Participants enrolled in TICH-2 may be enrolled in RESTART after 21 days.
2. Patient does not receive randomised treatment
3. Failure to complete SAEs where appropriate
4. Failure to complete outcomes where appropriate
5. Follow-up assessments are performed (as opposed to submitted) outside the specified time as shown below:

- 2-day follow-up: >2 days past the due date
- 7-day follow-up: >7 days past the due date
- Discharge and Death form: >30 days past the due date
- 90-day follow up: >30 days past the due date
- 365 day follow up: > 30 days past the due date

Suspected protocol violations are reported online by investigators and then adjudicated, by the trial management committee, on definition and investigators adherence to the protocol. For example, if a participant had died after giving brief consent, but before full written consent could be obtained, then this does not constitute a protocol violation as the investigators adhered to the protocol and the interpretation of trial delivery was not significantly affected.

### 2.2 Oxfordshire Community Stroke Project (OCSP) classification

The Oxfordshire Community Stroke Project (OCSP) classification will be calculated from the baseline NIHSS, using the following algorithm [22].

| OCSP classification | NIHSS questions [23] |
| --- | --- |
| TACS (Total Anterior Circulation Stroke) | All three of:   1. Unilateral weakness (and/or sensory deficit) of face, arm and leg – Q4, 5, 6, 8 2. Homonymous hemianopia – Q3 3. Higher cerebral dysfunction (dysphasia, visuospatial disorder) – Q1b, 1c, 3, 9, 11 |
| PACS (Partial Anterior Circulation Syndrome) | Two of:   1. Unilateral weakness (and/or sensory deficit) of face, arm and leg – Q4, 5, 6, 8 2. Homonymous hemianopia – Q3 3. Higher cerebral dysfunction (dysphasia, visuospatial disorder) – Q1b, 1c, 3, 9, 11 |
| POCS (POsterior Circulation Syndrome) | One of:   1. Cerebellar or brainstem syndromes – location of infra-tentorial 2. Loss of consciousness – Q1a, 2 3. Isolated homonymous hemianopia – Q3 |
| LACS (LACunar Syndrome) | One of:   1. Unilateral weakness (and/or sensory deficit) of face and arm, arm and leg or all three – Q4, 5, 6, 8 2. Pure sensory stroke – Q8 3. Ataxic hemiparesis – Q7 |

### 2.3 Euroqol-5D

Euroqol-5 Dimensions Health Utility State (EQ-5D HUS) is a measure of health state using five dimensions: mobility, self-care, usual activities, pain/discomfort and anxiety/depression. Each of these dimensions has three levels on which the patient can score themselves; level one means they have no problems, level two means some problems and level three means they have severe problems [24]. All five of the questions must be answered for a total to be calculated, using the following algorithm [25].

|  | **Weight** |
| --- | --- |
| Full health | 1 |
| At least one 2 or 3 | -0.081 |
| At least one 3 | -0.269 |
| Mobility |  |
| - Score 2 | -0.069 |
| - Score 3 | -0.314 |
| Self-care |  |
| - Score 2 | -0.104 |
| - Score 3 | -0.214 |
| Usual activities |  |
| - Score 2 | -0.036 |
| - Score 3 | -0.094 |
| Pain/discomfort |  |
| - Score 2 | -0.123 |
| - Score 3 | -0.386 |
| Anxiety/depression |  |
| - Score 2 | -0.071 |
| - Score 3 | -0.236 |

### 2.4 Haematoma volume

Haematoma expansion will be determined as follows: ICH haematoma volume (HV), expressed in millilitres, will be calculated on the CT scans performed at baseline (pre-enrolment) and at 24 hours post-randomisation using the ABC/2 method and semi-automated segmentation methods (ITK-SNAP 3.4.0). The measurements derived from the semi-automated segmentation method will be used for analysis of the haematoma expansion outcome. This is to ensure uniformity with intraventricular haemorrhage and perihaematomal oedema volumes, which are measured using semi-automated segmentation only. Absolute growth of haematoma will be calculated as HV_24hours_ - HV_baseline_. Relative growth will be calculated as (HV_24hours_ - HV_baseline_) / HV_baseline_, and will be expressed as a percentage. Haematoma expansion will be defined as an absolute growth of >6 ml or a relative growth of >33%; these thresholds have been shown to have positive predictive value of poor outcome (mRS 4-6) of 81% and 68%, respectively [26].

Intraventricular haematoma (IVH) volume will be calculated by semi-automated segmentation on baseline and 24 hour post randomisation CT scans, with absolute and relative haematoma growth calculated as above. ICH and IVH volumes will be added to give total haematoma volumes, allowing calculation of growth of total haematoma as above. Perihaematomal oedema (defined as the tissue immediately adjacent to the haematoma with reduced attenuation compared normal-appearing brain parenchyma) will be measured using semi-automated segmentation; absolute and relative growth will be calculated as above. All measurements will be carried out by trained image analysts for whom performance characteristics (intra- and inter-observer reliability measures) will be calculated.

# Appendix C – Tables and figures in primary publications

## Table 1 – Baseline characteristics

|  | **All** | **TA** | **Control** |
| --- | --- | --- | --- |
| Patients randomised |  |  |  |
| Age (years)*, Mean (SD) {range} |  |  |  |
| >70 years, (%) |  |  |  |
| Sex*, Male (%) |  |  |  |
| Ethnic origin, (%) |  |  |  |
| White |  |  |  |
| Black |  |  |  |
| South Asian |  |  |  |
| East Asian^†^ |  |  |  |
| Other Asian |  |  |  |
| Other |  |  |  |
| Time from onset to randomisation (hours)*, Median [IQR] {range} |  |  |  |
| >3 hours (%) |  |  |  |
| >4.5 hours (%) |  |  |  |
| History of antiplatelet therapy on admission*, (%) |  |  |  |
| History of statin use prior to admission, (%) |  |  |  |
| History of previous ischaemic stroke or TIA, (%) |  |  |  |
| History of IHD, (%) |  |  |  |
| History of thromboembolism, (%) |  |  |  |
| Pre-stroke mRS, Median [IQR] {range} |  |  |  |
| >2, (%) |  |  |  |
| GCS, Mean (SD) {range} |  |  |  |
| <8, (%) |  |  |  |
| NIHSS score*, Mean (SD) {range} |  |  |  |
| >13, (%) |  |  |  |
| SBP (mmHg)*, Mean (SD) {range} |  |  |  |
| >170 mmHg, (%) |  |  |  |
| DBP (mmHg), Mean (SD) {range} |  |  |  |
| OCSP, (%) |  |  |  |
| TACS |  |  |  |
| PACS |  |  |  |
| POCS |  |  |  |
| LACS |  |  |  |
| Intra-ventricular haemorrhage*, (%) |  |  |  |
| Advanced imaging performed, (%) |  |  |  |
| Spot positive |  |  |  |
| Spot negative |  |  |  |
| Haematoma location, (%) |  |  |  |
| Supra-tentorial Lobar |  |  |  |
| Supra-tentorial Deep |  |  |  |
| Infra-tentorial |  |  |  |
| Combination of above locations |  |  |  |
| Haematoma volume |  |  |  |
| Intracerebral haematoma volume (ml), Mean (SD) {range} |  |  |  |

*minimisation criteria

^†^Including South East Asia

SD: standard deviation; IQR: interquartile range; TIA: transient ischaemic attack; IHD: ischaemic heart disease; mRS: modified rankin scale; GCS: Glasgow coma scale; NIHSS: national institutes of health stroke scale; SBP: systolic blood pressure; DBP: diastolic blood pressure; OSCP: Oxfordshire community stroke project; TACS: total anterior circulation stroke; PACS: partial anterior circulation syndrome; POCS: posterior circulation syndrome; LACS: lacunar syndrome

## Table 2 – Compliance

|  | **All** | **TA** | **Control** | **p** |
| --- | --- | --- | --- | --- |
| **Number of participants randomised** |  |  |  |  |
| All randomised treatment received as allocated |  |  |  |  |
| Some randomised treatment received as allocated |  |  |  |  |
| No randomised treatment received as allocated |  |  |  |  |
| Time from randomisation until treatment (hours), median [IQR] {range} |  |  |  |  |

## Table 3 – Outcomes

|  | | | | | **Adjusted** | | **Unadjusted** | |
| --- | --- | --- | --- | --- | --- | --- | --- | --- |
|  | **n** | **All** | **TA** | **Control** | **OR/MD/HR (95% CI)** | **p** | **OR/MD/HR (95% CI)** | **p** |
| Total number of participants randomised |  |  |  |  |  |  |  |  |
|  | | | | | | | | |
| **Primary outcome, day 90** |  |  |  |  |  |  |  |  |
| Total number of patients with outcome (mRS, /6) |  |  |  |  |  |  |  |  |
| 0 |  |  |  |  | OLR OR |  |  |  |
| 1 |  |  |  |  |  |  |  |  |
| 2 |  |  |  |  |  |  |  |  |
| 3 |  |  |  |  |  |  |  |  |
| 4 |  |  |  |  |  |  |  |  |
| 5 |  |  |  |  |  |  |  |  |
| 6 (death) |  |  |  |  |  |  |  |  |
|  | | | | | | | | |
| **Sensitivity analyses, day 90** |  |  |  |  |  |  |  |  |
| mRS, median [IQR] {range} |  |  |  |  |  |  |  |  |
| mRS, mean (SD) |  |  |  |  | MLR MD |  |  |  |
| mRS ≥ 3, n (%) |  |  |  |  | BLR OR |  |  |  |
|  | | | | | | | | |
| **Secondary outcomes** |  |  |  |  |  |  |  |  |
| **Day 7** |  |  |  |  |  |  |  |  |
| Death by day 7 |  |  |  |  | BLR OR |  |  |  |
| NIHSS score (/40) |  |  |  |  | MLR MD |  |  |  |
| **Day 90** |  |  |  |  |  |  |  |  |
| Death by day 90 |  |  |  |  | CPHR HR |  |  |  |
| EQ-5D HUS (/1) |  |  |  |  | MLR MD |  |  |  |
| EQ-VAS ( /100) |  |  |  |  | MLR MD |  |  |  |
| Barthel Index ( /100) |  |  |  |  | MLR MD |  |  |  |
| TICS-M (/39) |  |  |  |  | MLR MD |  |  |  |
| ZDS (/100) |  |  |  |  | MLR MD |  |  |  |
| Global difference |  |  |  |  | Mann-Whitney |  |  |  |
| **Discharge** |  |  |  |  |  |  |  |  |
| Length of stay in hospital, time until discharge* |  |  |  |  | CPHR HR |  |  |  |
| Time well at home (days) |  |  |  |  | MLR MD |  |  |  |
| **Disposition at discharge** |  |  |  |  | OLR OR |  |  |  |
| Home alone |  |  |  |  | BLR OR |  |  |  |
| Home with family/ carers |  |  |  |  | BLR OR |  |  |  |
| … |  |  |  |  |  |  |  |  |
|  |  |  |  |  |  |  |  |  |
| **Haematoma** |  |  |  |  |  |  |  |  |
| Change in volume from baseline to 24 hours |  |  |  |  | MLR MD |  |  |  |
| Participants with haematoma expansion^†^, n (%) |  |  |  |  | BLR OR |  |  |  |

*If a participant died before they were discharged they will be censored in the analysis.

^†^Haematoma expansion as defined in appendix B, section 2.4

SD: standard deviation; IQR: interquartile range; OR: odds ratio; MD: mean difference; HR: hazard ratio; mRS: modified rankin scale; GCS: Glasgow coma scale; NIHSS: national institutes of health stroke scale; EQ-5D HUS: EuroQol 5-dimensions health utility status; EQ-VAS: EuroQol visual analogue scale; TICS-m: telephone interview for cognitive status-modified; ZDS: Zung depression scale

## Table 4 – Serious adverse events

This table gives details on the serious adverse events experienced up until each participant’s day 7 follow-up; safety outcomes and death are collected up until day 90. The numbers given in each group are per participant, as opposed to the total number of events. The by day section is a cumulative count up to day 90. The severity counts are ordinal; each participant with an event will be counted only once, with their most severe event being counted. The by site section will be given for number of participants in each row; if someone had an event in both the cardiovascular and nervous system categories then they would appear twice, once in each of these categories. As well as the site categories, each type of SAE experiences will also be given. Comparisons will be done using binary logistic regression.

|  | **Median time to event** | **All** | **TA** | **Control** | **p** |
| --- | --- | --- | --- | --- | --- |
| By day 2 |  |  |  |  |  |
| By day 7 |  |  |  |  |  |
| By day 90 |  |  |  |  |  |
|  |  |  |  |  |  |
| **By severity** |  |  |  |  |  |
| Severe, fatal |  |  |  |  |  |
| Severe, not fatal |  |  |  |  |  |
| Moderate |  |  |  |  |  |
| Mild |  |  |  |  |  |
|  |  |  |  |  |  |
| **Safety outcomes** |  |  |  |  |  |
|  |  |  |  |  |  |
|  |  |  |  |  |  |
|  |  |  |  |  |  |
| **SAEs by site** |  |  |  |  |  |
| Blood and lymphatic system disorders |  |  |  |  |  |
|  |  |  |  |  |  |
| Cardiac disorders |  |  |  |  |  |
|  |  |  |  |  |  |
| Gastrointestinal disorders |  |  |  |  |  |
|  |  |  |  |  |  |
| General disorders and administration site conditions |  |  |  |  |  |
|  |  |  |  |  |  |
| Hepatobiliary disorders |  |  |  |  |  |
|  |  |  |  |  |  |
| Immune system disorders |  |  |  |  |  |
|  |  |  |  |  |  |
| Infections and infestations |  |  |  |  |  |
|  |  |  |  |  |  |
| Injury, poisoning and procedural complications |  |  |  |  |  |
|  |  |  |  |  |  |
| Metabolism and nutrition disorders |  |  |  |  |  |
|  |  |  |  |  |  |
| Musculoskeletal and connective tissue disorders |  |  |  |  |  |
|  |  |  |  |  |  |
| Neoplasms benign, malignant and unspecified (incl. cysts and polyps) |  |  |  |  |  |
|  |  |  |  |  |  |
| Nervous system disorders |  |  |  |  |  |
|  |  |  |  |  |  |
| Psychiatric disorders |  |  |  |  |  |
|  |  |  |  |  |  |
| Renal and urinary disorders |  |  |  |  |  |
|  |  |  |  |  |  |
| Respiratory, thoracic and mediastinal disorders |  |  |  |  |  |
|  |  |  |  |  |  |
| Skin and subcutaneous tissue disorders |  |  |  |  |  |
|  |  |  |  |  |  |
| Vascular disorders |  |  |  |  |  |
|  |  |  |  |  |  |
| Miscellaneous |  |  |  |  |  |
|  |  |  |  |  |  |

## Table 5 – Outcome by subgroups

This table will be presented as a forest plot in the final publication.

| Variable | N | TA | Control | OR (95% CI) | Interaction p |
| --- | --- | --- | --- | --- | --- |
| Onset to randomisation |  |  |  |  |  |
| ≤3 hours |  |  |  |  |  |
| >3 hours |  |  |  |  |  |
| Age |  |  |  |  |  |
| <70 |  |  |  |  |  |
| >70 |  |  |  |  |  |
| Sex |  |  |  |  |  |
| Female |  |  |  |  |  |
| Male |  |  |  |  |  |
| Systolic blood pressure |  |  |  |  |  |
| ≤170 mmHg |  |  |  |  |  |
| >170 mmHg |  |  |  |  |  |
| NIHSS score |  |  |  |  |  |
| ≤15 |  |  |  |  |  |
| >15 |  |  |  |  |  |
| Intra-ventricular haemorrhage |  |  |  |  |  |
| Yes |  |  |  |  |  |
| No |  |  |  |  |  |
| History of antiplatelet therapy |  |  |  |  |  |
| Yes |  |  |  |  |  |
| No |  |  |  |  |  |
| CTA |  |  |  |  |  |
| Yes |  |  |  |  |  |
| No |  |  |  |  |  |
| Haematoma location |  |  |  |  |  |
| Supra-tentorial Lobar |  |  |  |  |  |
| Supra-tentorial Deep |  |  |  |  |  |
| Ethnicity |  |  |  |  |  |
| White |  |  |  |  |  |
| Other |  |  |  |  |  |

## Figure 1 – Trial flow diagram

**Number of patients randomised**

n=

**Tranexamic acid**

Number allocated, n=

Baseline data completed, n=

**Placebo**

Number allocated, n=

Baseline data completed, n=

**Day 2 follow-up**

Death by day 2, n=

**Day 2 assessment completed, n=**

Day 2 assessment missing

Patient withdrawn, n=

Other reason, n=

**Day 2 follow-up**

Death by day 2, n=

**Day 2 assessment completed, n=**

Day 2 assessment missing

Patient withdrawn, n=

Other reason, n=

**Day 7 follow-up**

Death by day 7, n=

**Day 7 assessment completed, n=**

Day 7 assessment missing

Patient withdrawn, n=

Other reason, n=

**Day 7 follow-up**

Death by day 7, n=

**Day 7 assessment completed, n=**

Day 7 assessment missing

Patient withdrawn, n=

Other reason, n=

**Day 90 follow-up (end of follow-up)**

**Day 90 mRS available (inc. deaths), n=**

Death by day 90, n=

**Day 90 mRS assessment missing**

Patient lost to follow-up, n=

Patient refused, n=

Patient withdrawn, n=

Other reason, n=

**Day 90 follow-up (end of follow-up)**

**Day 90 mRS available (inc. deaths), n=**

Death by day 90, n=

**Day 90 mRS assessment missing**

Patient lost to follow-up, n=

Patient refused, n=

Patient withdrawn, n=

Other reason, n=

**Day 365 follow-up**

**Day 365 mRS available (inc. deaths), n=**

Death by day 365, n=

**Day 365 mRS assessment missing**

Patient lost to follow-up, n=

Patient refused, n=

Patient withdrawn, n=

Other reason, n=

**Day 365 follow-up**

**Day 365 mRS available (inc. deaths), n=**

Death by day 365, n=

**Day 365 mRS assessment missing**

Patient lost to follow-up, n=

Patient refused, n=

Patient withdrawn, n=

Other reason, n=

## Figure 2 – Shift plot of mRS at day 90 by treatment group

This graph gives an unadjusted visual representation of the relationship between treatment and day 90 mRS scores. The below graph was made as an example, using dummy treatments and scores.

# Appendix D – Secondary publications

## 1 Published

***Protocol publication***

This outlines the rationale, methods and design of the TICH-2 study.

## 2 Submitted

None submitted.

## 3 In preparation

#### Statistical analysis plan (this publication)

## 4 Planned (not a definitive list)

#### Baseline paper

This will contain information on all of the randomisation and baseline variables, including a more detailed list of the ethnicities of the participants enrolled.

#### Safety outcomes

This will include more detailed information on each of the safety outcomes collected in the trial; their relationship to the mRS, and other outcomes, at day 90; and time-to-event analyses.

**Radiological evidence of on-going bleeding**

Haematoma expansion is compared between allocated treatment groups (TXA vs. placebo) in patient with and without radiological signs of ongoing bleeding on admission. Ongoing bleeding is defined as presence of spot sign on CT-angiography and/or extravasation of contrast on post-contrast sequences. Further, haematoma expansion is compared between allocated treatment groups (TXA vs. placebo) in patients with and without hypodensities within the acute haematoma on non-contrast CT. This analysis is planned in order to describe the effect of on-going bleeding on treatment efficacy (the PATCH-it protocol).

#### Other outcomes

This will include more detailed information on outcomes collected from days 2, 7 and 90; including deaths, NIHSS scores and GCS scores. It will also detail other possible relationships with the mRS and outcomes/ SAEs.

#### Emergency consent

This paper will detail the consent processes used in TICH-2.

#### Day 365 sub study

The day 365 paper will go into a more detailed analysis of the outcomes at day 365.

**Example table – Day 365 outcomes**

|  | | | | | **Adjusted** | |
| --- | --- | --- | --- | --- | --- | --- |
|  | **n** | **All** | **TA** | **Control** | **OR (95% CI)** | **2p** |
| **Day 365, n (%)** |  |  |  |  |  |  |
| Death by day 365, (%) |  |  |  |  | BLR OR |  |
| Death between day 90 and day 365, (%) |  |  |  |  | BLR OR |  |
| EQ-5D HUS (/1) |  |  |  |  | MLR MD |  |
| EQ-VAS ( /100) |  |  |  |  | MLR MD |  |
| Barthel index ( /100) |  |  |  |  | MLR MD |  |
| TICS-M (/39) |  |  |  |  | MLR MD |  |
| ZDS (/100) |  |  |  |  | MLR MD |  |
| Ordinal mRS, (%) |  |  |  |  |  |  |
| 0 |  |  |  |  | OLR OR |  |
| 1 |  |  |  |  |  |  |
| 2 |  |  |  |  |  |  |
| 3 |  |  |  |  |  |  |
| 4 |  |  |  |  |  |  |
| 5 |  |  |  |  |  |  |
| 6 |  |  |  |  |  |  |
| mRS, median [IQR] {range} |  |  |  |  |  |  |
| mRS, mean (SD) |  |  |  |  | MLR MD |  |
| mRS > 3, (%) |  |  |  |  | BLR OR |  |

#### Individual patient data meta-analysis (IPDMA) of all tranexamic acid in ICH studies

This will collect individual patient data from studies that looked at using TXA for an ICH.

# References

1. Jonjev, Z.S., et al., *Prostacyclin reduces incidence of myocardial damage after coronary endarterectomy.* Annals of Thoracic Surgery, 2004. **78**(4): p. 1299-303.

2. Cui, J., et al., *Laboratory and non-laboratory-based risk prediction models for secondary prevention of cardiovascular disease: the LIPID study.* European Journal of Cardiovascular Prevention & Rehabilitation, 2009. **16**(6): p. 660-8.

3. Weir, C.J. and K.R. Lees, *Comparison of stratification and adaptive methods for treatment allocation in an acute stroke clinical trial.* Stat.Med., 2003. **22**(5): p. 705-726.

4. TICH-2. *Trial documents*. 2016 4th October 2017]; Available from: <https://www.nottingham.ac.uk/~nszwww/tich-2/ShWrtq5IdxUu8LdpTzMfD2U3Uh4.php>.

5. Collaborators, C.-. *The importance of early treatment with tranexamic acid in bleeding trauma patients: an exploratory analysis of the CRASH-2 randomised controlled trial.* The Lancet, 2011. **377**(9771): p. 1096-1101. e2.

6. Du, F.-Z., et al., *The accuracy of spot sign in predicting hematoma expansion after intracerebral hemorrhage: a systematic review and meta-analysis.* PLoS ONE [Electronic Resource], 2014. **9**(12): p. e115777.

7. Del Giudice, A., et al., *Accuracy of the spot sign on computed tomography angiography as a predictor of haematoma enlargement after acute spontaneous intracerebral haemorrhage: a systematic review.* Cerebrovascular Diseases, 2014. **37**(4): p. 268-276.

8. Xi, G., R.F. Keep, and J.T. Hoff, *Mechanisms of brain injury after intracerebral haemorrhage.* The Lancet Neurology, 2006. **5**(1): p. 53-63.

9. Fogelholm, R., M. Nuutila, and A. Vuorela, *Primary intracerebral haemorrhage in the Jyväskylä region, central Finland, 1985-89: incidence, case fatality rate, and functional outcome.* Journal of Neurology, Neurosurgery & Psychiatry, 1992. **55**(7): p. 546-552.

10. Andrews, B.T., et al., *The effect of intracerebral hematoma location on the risk of brain-stem compression and on clinical outcome.* Journal of neurosurgery, 1988. **69**(4): p. 518-522.

11. van Asch, C.J., et al., *Incidence, case fatality, and functional outcome of intracerebral haemorrhage over time, according to age, sex, and ethnic origin: a systematic review and meta-analysis.* The Lancet Neurology, 2010. **9**(2): p. 167-176.

12. Hu, H.H., et al., *Incidence of stroke in Taiwan.* Stroke, 1992. **23**(9): p. 1237-41.

13. Sun, Z., et al., *An epidemiological survey of stroke among rural Chinese adults results from the Liaoning province.* Int J Stroke, 2013. **8**(8): p. 701-6.

14. Sudlow, C.L. and C.P. Warlow, *Comparable studies of the incidence of stroke and its pathological types: results from an international collaboration. International Stroke Incidence Collaboration.* Stroke, 1997. **28**(3): p. 491-9.

15. Thrift, A.G., et al., *Incidence of the major stroke subtypes: initial findings from the North East Melbourne stroke incidence study (NEMESIS).* Stroke, 2001. **32**(8): p. 1732-8.

16. Inagawa, T., et al., *Primary intracerebral hemorrhage in Izumo City, Japan: incidence rates and outcome in relation to the site of hemorrhage.* Neurosurgery, 2003. **53**(6): p. 1283-1298.

17. Kimura, Y., et al., *Demographic study of first-ever stroke and acute myocardial infarction in Okinawa, Japan.* Internal Medicine, 1998. **37**(9): p. 736-745.

18. Feigin, V., et al., *Ethnic disparities in incidence of stroke subtypes: Auckland Regional Community Stroke Study, 2002-2003.* Lancet Neurol, 2006. **5**(2): p. 130-9.

19. Wei, L. and J. Lachin, *Two-sample asymptotically distribution-free tests for incomplete multivariate observations.* Journal of the American Statistical Association, 1984. **79**(387): p. 653-661.

20. Lachin, J.M., *Applications of the Wei-Lachin multivariate one-sided test for multiple outcomes on possibly different scales.* PLoS ONE [Electronic Resource], 2014. **9**(10): p. e108784.

21. The Optimising Analysis of Stroke Trials (OAST) Collaboration, *Should stroke trials adjust functional outcome for baseline prognostic factors?* Stroke, 2009. **40**: p. 888-894.

22. Med Quarterly. *Oxford Stroke Classification*. [cited 2015 13 July]; Available from: <http://www.medquarterly.com/mq88/MQPDF/MM/OxfordStrokeClassification.pdf>.

23. Lyden, P., et al., *Improved reliability of the NIH stroke scale using video training.* Stroke, 1994. **25**: p. 2220-2226.

24. Rabin, R., M. Oemar, and M. Oppe, *EQ-5D-3L User Guide Basic Information on How to Use the EQ-5D-3L Instrument.* Rotterdam: EuroQol Group, 2011.

25. Whynes, D.K., et al., *Testing for differential item functioning within the EQ-5D.* Medical Decision Making, 2012: p. 0272989X12465016.

26. Dowlatshahi, D., et al., *Small intracerebral haemorrhages are associated with less haematoma expansion and better outcomes.* International Journal of Stroke, 2011. **6**(3): p. 201-206.
